# Inequalities in the use of secondary prevention of cardiovascular disease by socioeconomic status: evidence from the PURE observational study

**DOI:** 10.1016/S2214-109X(18)30031-7

**Published:** 2018-02-09

**Authors:** Adrianna Murphy, Benjamin Palafox, Owen O'Donnell, David Stuckler, Pablo Perel, Khalid F AlHabib, Alvaro Avezum, Xiulin Bai, Jephat Chifamba, Clara K Chow, Daniel J Corsi, Gilles R Dagenais, Antonio L Dans, Rafael Diaz, Ayse N Erbakan, Noorhassim Ismail, Romaina Iqbal, Roya Kelishadi, Rasha Khatib, Fernando Lanas, Scott A Lear, Wei Li, Jia Liu, Patricio Lopez-Jaramillo, Viswanathan Mohan, Nahed Monsef, Prem K Mony, Thandi Puoane, Sumathy Rangarajan, Annika Rosengren, Aletta E Schutte, Mariz Sintaha, Koon K Teo, Andreas Wielgosz, Karen Yeates, Lu Yin, Khalid Yusoff, Katarzyna Zatońska, Salim Yusuf, Martin McKee

**Affiliations:** a Centre for Global Chronic Conditions, London School of Tropical Medicine, London, UK; b Erasmus School of Economics, Erasmus University Rotterdam, Rotterdam, Netherlands; c Faculty of Economics and Business, University of Lausanne, Lausanne, Switzerland; d Department of Policy Analysis and Public Management, Bocconi University, Milan, Italy; e Department of Cardiac Sciences, King Fahad Cardiac Center, College of Medicine, King Saud University, Riyadh, Saudi Arabia; f Institute of Cardiology, University of Santo Amaro, Sao Paulo, Brazil; g State Key Laboratory of Cardiovascular Disease, Fuwai Hospital, National Center for Cardiovascular Disease, Peking Union Medical College and Chinese Academy of Medical Sciences, Beijing, China; h Department of Physiology, College of Health Sciences, University of Zimbabwe, Harare, Zimbabwe; i The University of Sydney and The George Institute for Global Health, Camperdown, NSW, Australia; j Ottawa Hospital Research Institute, OMNI Research Group, Clinical Epidemiology Program, Ottawa, ON, Canada; k Institut Universitaire de Cardiologie et Pneumologie de Québec, Québec City, QC, Canada; l University of the Philippines—Manila, Manila, Philippines; m Estudios Clínicos Latinoamérica (ECLA) International, Rosario, Santa Fe, Argentina; n Nisa Hastanesi, Fatih, Istanbul, Turkey; o Department of Community Health, UKM Medical Centre, University Kebangsaan Malaysia, Kuala Lumpur, Malaysia; p Departments of Community Health Sciences and Medicine, Aga Khan University, Karachi, Pakistan; q Isfahan Cardiovascular Research Center, Cardiovascular Research Institute, Isfahan University of Medical Sciences, Chamran Hospital, Isfahan, Iran; r Department of Public Health Sciences, Loyola Medical Center, Maywood, IL, USA; s Universidad de La Frontera, Temuco, Chile; t Simon Fraser University, Burnaby, BC, Canada; u Fundación Oftalmológica de Santander-FOSCAL—FOSCAL Internacional, Floridablanca, Santander, Colombia; v Madras Diabetes Research Foundation and DrMohan's Diabetes Specialities Centre, Gopalapuram, Chennai, India; w Dubai Health Authority, Dubai, United Arab Emirates; x St John's Medical College and Research Insitute, Bangalore, India; y School of Public Health, University of the Western Cape, Cape Town, Western Cape Province, South Africa; z Population Health Research Institute, McMaster University, C2-106 DBCVSRI Hamilton General Hospital, Hamilton, ON, Canada; aa Department of Molecular and Clinical Medicine, Sahlgrenska Academy, University of Gothenburg, and Sahlgrenska University Hospital/Östra, Göteborg, Sweden; ab South African Medical Research Council Unit for Hypertension and Cardiovascular Disease, Hypertension in Africa Research Team (HART), North-West University, Potchefstroom, South Africa; ac Independent University, Bangladesh, Dhaka, Bangladesh; ad University of Ottawa Department of Medicine, Ottawa, ON, Canada; ae Department of Medicine, Queen's University, Kingston, ON, Canada; af Universiti Teknologi MARA, Selyang Campus, Selayang, Selangor and UCSI University, Cheras, Malaysia; ag Department of Social Medicine, Medical University, Wrocław, Poland

## Abstract

**Background:**

There is little evidence on the use of secondary prevention medicines for cardiovascular disease by socioeconomic groups in countries at different levels of economic development.

**Methods:**

We assessed use of antiplatelet, cholesterol, and blood-pressure-lowering drugs in 8492 individuals with self-reported cardiovascular disease from 21 countries enrolled in the Prospective Urban Rural Epidemiology (PURE) study. Defining one or more drugs as a minimal level of secondary prevention, wealth-related inequality was measured using the Wagstaff concentration index, scaled from −1 (pro-poor) to 1 (pro-rich), standardised by age and sex. Correlations between inequalities and national health-related indicators were estimated.

**Findings:**

The proportion of patients with cardiovascular disease on three medications ranged from 0% in South Africa (95% CI 0–1·7), Tanzania (0–3·6), and Zimbabwe (0–5·1), to 49·3% in Canada (44·4–54·3). Proportions receiving at least one drug varied from 2·0% (95% CI 0·5–6·9) in Tanzania to 91·4% (86·6–94·6) in Sweden. There was significant (p<0·05) pro-rich inequality in Saudi Arabia, China, Colombia, India, Pakistan, and Zimbabwe. Pro-poor distributions were observed in Sweden, Brazil, Chile, Poland, and the occupied Palestinian territory. The strongest predictors of inequality were public expenditure on health and overall use of secondary prevention medicines.

**Interpretation:**

Use of medication for secondary prevention of cardiovascular disease is alarmingly low. In many countries with the lowest use, pro-rich inequality is greatest. Policies associated with an equal or pro-poor distribution include free medications and community health programmes to support adherence to medications.

**Funding:**

Full funding sources listed at the end of the paper (see Acknowledgments).

## Introduction

The UN Sustainable Development Goal 3 aims for a 30% reduction in non-communicable diseases by 2030.^1^ This aim will require substantial reductions in cardiovascular disease.^2^, ^3^, ^4^ Secondary prevention of recurrent myocardial infarction and stroke among those with known cardiovascular disease can reduce cardiovascular mortality substantially.^2^, ^3^ The WHO Global Monitoring Framework for Non-Communicable Diseases aims for at least 50% coverage of those eligible with drug therapy and counselling by 2025.^5^

The cost-effectiveness of secondary prevention of cardiovascular disease, coupled with lifestyle changes, has long been established.^4^, ^6^ Yet, the Prospective Urban Rural Epidemiology (PURE) study has demonstrated that within groups of countries categorised by income (low, lower-middle, upper-middle, and high), average use of drug treatment is low, particularly in the low-income countries,^3^ treatment is unavailable or unaffordable for many people,^7^ and its use is associated with wealth in south Asia^8^ and South America.^9^ Thus far, however, there are no comparisons of rates or extent of inequalities in the use of secondary prevention of cardiovascular disease across countries using consistent methods, and what data do exist are almost all from high-income countries. A recent systematic review^10^ identified ten studies that reported lower treatment rates among patients with lower social economic status. Only two studies looked beyond high-income countries, both set in China. One, which developed a composite measure of socioeconomic status based on education, income, occupation, and access to medical insurance, reported 43% lower use of aspirin and over 70% lower use of antiplatelet agents, statins, and β-blockers among patients with lower socioeconomic status.^11^ The other study examined inequalities by age, comparing patients older and younger than 65 years, finding lower use of secondary prevention in the former.^12^ Evidence on the level and distribution of secondary prevention at the country level is crucial for designing national health system policies that can reduce premature cardiovascular disease mortality and morbidity.

**Research in context**

**Evidence before this study**

A systematic review of papers published between 1996 and 2015, in English and German, on socioeconomic inequalities in access to treatment for cardiovascular disease found 18 papers on secondary prevention, ten of which reported lower uptake in patients with lower socioeconomic status. Other research within the PURE study had examined inequalities in use of secondary prevention, but only using data combined from groups of countries defined by income or geographical region.

**Added value of this study**

This paper presents the first country-specific data on inequalities in use of secondary prevention for cardiovascular disease in countries at all levels of development and in all parts of the world. It reveals marked cross-country differences in the extent to which there is equitable utilisation among those with differing levels of wealth, and it points to potential explanations of these differences.

**Implications of all the available evidence**

Use of secondary prevention for cardiovascular disease is alarmingly low. Many of the countries with the lowest overall use also have the greatest pro-rich inequality in use. Countries with a pro-poor distribution have policies, such as free medications and community health programmes, to support adherence to medications that might improve secondary prevention of cardiovascular disease among the poor.

The objectives of this analysis are to use the PURE study data to produce the first estimates of socioeconomic inequality in the use of secondary prevention for cardiovascular disease within 21 countries at varying levels of development and to investigate health system factors that might be correlated with this inequality. We hypothesise that pro-rich inequality in the use of secondary prevention medicine for cardiovascular disease exists in some countries, and that this is associated with health system factors such as affordability of medicines and public expenditure on health care.

## Methods

### The PURE study

PURE is a large international study of the incidence, mortality, and risk factors associated with non-communicable diseases,^13^ and includes individuals from urban and rural communities in 21 countries: Canada, Sweden, United Arab Emirates, Saudi Arabia, Argentina, Brazil, Chile, Malaysia, Poland, South Africa, Turkey, China, the Philippines, Colombia, Iran, the occupied Palestinian territory, Bangladesh, India, Pakistan, Zimbabwe, and Tanzania (in order of income, using 2006 per capita gross domestic product [GDP] when the study was initiated).

Data collection in PURE has been described in detail elsewhere.^13^ Briefly, in each country, communities were selected to achieve a mix of rural and urban populations, while ensuring feasibility of data collection (eg, processing blood samples) and long-term follow-up. Households were selected to be broadly representative of the sociodemographic composition of communities. Although not designed to be nationally representative, the sociodemographic characteristics and death rates of the samples of the first 17 participating countries were similar to their national populations.^14^ Within each selected household, all individuals aged 35–70 years were eligible to participate. Each participant was interviewed using a standardised questionnaire and had a medical examination. Data included sociodemographic characteristics, biometrics, lifestyle, and behaviour, cardiovascular disease risk factors, health history, and the use of medications.^13^ The years of data collection and the response rates for each country are in the appendix (pp 2, 3).

Ethics approval was acquired in each country from the local institutional ethical review board. All participants in the PURE study signed an informed consent form.

### Procedures

Our population of interest comprises 8492 participants with known cardiovascular disease at recruitment. Cardiovascular disease was defined as self-reported myocardial infarction, coronary artery bypass graft surgery or percutaneous coronary angioplasty, angina, or stroke. Self-reports were verified against medical or hospital records in 455 reported events, with a confirmation rate of 89%.^3^ Use of medicines was defined by patient responses to the question: “List all the medications you are currently consuming at least once a week for the last month”. Self-reports of medicines being used were verified by asking patients to show the field workers their prescriptions or medical documents. We first investigated use of an optimal drug regimen for secondary prevention of cardiovascular disease, which includes an antiplatelet drug (aspirin, clopidogrel, or other antiplatelet), cholesterol-lowering drug (statin, ezetimibe, or other cholesterol-lowering drug), a β-blocker, and an angiotensin-converting-enzyme inhibitor or angiotensin-receptor blocker. Because the number of individuals with cardiovascular disease using the four-drug regimen was very low in many countries, we examined inequality in the use of one or more drugs, which indicates a minimal level (although inadequate) secondary prevention of cardiovascular disease. Results for the use of two or more drugs are presented in the appendix (p 8).

Following asset-based approaches for measuring wealth employed in the Demographic and Health Surveys,^15^ the PURE study collected data on household possessions, including electricity supply and ownership of an automobile, other four-wheel vehicle, computer, television, motorbike, livestock, refrigerator, washing machine, stereo, bicycle, kitchen mixer, telephone, land or real estate, and kitchen window.^15^ We used these data to generate an asset-based wealth index using principal components analysis within each country. This index places households within each country-specific sample on a continuous scale of relative wealth from poorest to richest.^16^ The index standardises the measurement of relative wealth across countries and enables meaningful cross-country comparisons.^17^ The distribution of wealth index scores for each country is shown in the appendix (p 4).

### Statistical analysis

We provide an initial estimation of socioeconomic inequality in use of secondary prevention drugs for cardiovascular disease by comparing rates of medication use across wealth index tertiles of respondents with cardiovascular disease within each country. Rates were standardised for age and sex using logistic regression including a random effect to account for clustering at the community level.

Our measure of inequality in use over the entire socioeconomic distribution was the concentration index.^18^ The concentration index is twice the covariance between a binary indicator of medication and (fractional) rank in the country-specific distribution of the wealth index (ie, 1/*N* for poorest, …, *N*/*N* for richest), divided by the mean rate of medication (for each country). If the use of secondary prevention drugs is not correlated with position in the wealth distribution, then the index is zero, indicating no socioeconomic inequality.

When applied to a binary variable, the range of the concentration index depends on the variable's mean, which confounds comparison of inequality across countries with different rates of medication. Further, the ordering of countries by degree of inequality can depend on whether the index is used to measure inequality in the use of medication or in the non-use of medication. We avoided these limitations by using Wagstaff's adjusted concentration index, which is simply the concentration index divided by 1 minus the mean rate of medication use (the same mean rate of medication used to calculate the concentration index).^19^ This index always lies in the range from −1 to 1, with a positive (or negative) value indicating disproportionate concentration of medication use among richer (or poorer) individuals. A value of 1 indicates that only the richest persons receive medication. We confirmed the robustness of our findings to estimating inequality using the alternative Erreygers' index^20^ that is less sensitive to very low and very high prevalence rates (appendix pp 5, 6).

Using the fact that a concentration index is a function of the covariance between an indicator of medicine use and (fractional) rank in the distribution of wealth, it is calculated (for each country) from a convenient least squares regression.^18^ Individual-level data were used. There was no grouping. SEs were obtained by the delta method applied to a non-linear function of the least squares coefficients, which is equal (by definition) to the concentration index, and adjusted for arbitrary correlation within communities, and heteroscedasticity of general form. Wagstaff's adjusted concentration index values were indirectly standardised for differences in age and sex (within each country) across the distribution of the wealth index. The model used to standardise the concentration indices is included in the appendix (p 7).

We hypothesised that increasing availability and affordability in a country will be associated with more equal use. We plotted values of Wagstaff's adjusted concentration index for each country against six measures related to availability and affordability of treatment in a country: overall rate of use of at least one secondary prevention drug, which acts as an indicator of availability and affordability combined; proportion of pharmacies in the community where all four secondary prevention medicines (ie, antiplatelet agent, statin, β-blocker, and one drug acting on the angiotensin system) are available; proportion of the sample for whom the price of all four secondary prevention drugs combined is unaffordable (defined as costing more than 20% of household income net of food expenditure^7^); gross national income per capita, adjusted for purchasing power parity; public expenditure on health as a proportion of GDP; and out-of-pocket expenditure on health. We use Kendall's rank correlation coefficient (Kendall's tau) to measure the strength and direction of the association between each of these variables and Wagstaff's adjusted concentration index. Kendall's tau coefficient (τ) is defined as: ([number of concordant pairs] – [number of discordant pairs]) / (*n*[*n *– 1] / 2). Data on overall use were obtained from the PURE study; data on availability and costs of medicines were from the linked Environmental Profile of a Community's Health instrument (details are included in the appendix, p 7);^21^ data on gross national income, public expenditure, and out-of-pocket expenditure were obtained from the World Bank Development Indicators database^22^ (using indicators for each country for the most recent year of data collection in that country; appendix, p 7). All analyses were done in Stata version 14.

### Role of the funding source

The funders of the study had no role in its design, data collection, data analysis, data interpretation, writing of the report, or in the decision to submit the paper for publication. The lead and senior authors (AM and MM) had full access to all the data in the study and all authors had final responsibility for the decision to submit for publication.

## Results

The countries with the highest rates of cardiovascular disease were China (7·4%, 95% CI 6·3–8·5) and Turkey (7·3%, 6·3–8·4). Countries with the lowest rates of cardiovascular disease were India (2·7%, 95% CI 2·2–3·1) and Bangladesh (2·7%, 2·2–3·4; table 1).

**Table 1:** Proportion of individuals with cardiovascular disease (CVD)* reported at entry in the PURE study countries

	**N**	**Age (years)**		**Women**			**Prevalence of CVD**		
		Mean (SD)	95% CI	n	%	95% CI	n	%	95% CI
Canada	10 388	53·4 (9·2)	52·9–53·9	5576	53·7%	52·3–55·1	606	5·8%	5·0–6·8
Sweden	4151	52·7 (9·0)	51·7–53·6	2193	52·8%	51·5–54·2	163	3·9%	3·1–5·0
United Arab Emirates	1499	48·3 (10·1)	45·9–50·7	981	65·4%	59·6–70·8	72	4·8%	3·0–7·7
Saudi Arabia	2047	46·5 (9·1)	45·9–47·1	882	43·1%	41·3–44·8	69	3·4%	2·7–4·2
Argentina	7511	51·2 (9·8)	50·8–51·5	4612	61·4%	58·8–64·0	293	3·9%	3·4–4·5
Brazil	6076	52·1 (9·4)	51·0–53·2	3345	55·1%	50·4–59·6	418	6·9%	6·1–7·7
Chile	3512	51·8 (9·8)	50·0–53·5	2313	65·9%	61·7–69·8	115	3·3%	2·0–5·2
Malaysia	15 567	51·6 (9·6)	50·8–52·5	8712	56·0%	54·3–57·6	435	2·8%	2·4–3·3
Poland	1976	54·4 (9·2)	53·4–55·5	1235	62·5%	60·1–64·8	131	6·6%	3·1–13·6
South Africa	4486	49·1 (9·7)	47·5–50·7	2969	66·2%	59·9–72·0	212	4·7%	3·1–7·1
Turkey	4231	50·0 (9·1)	49·4–50·5	2553	60·3%	57·9–62·8	308	7·3%	6·3–8·4
China	47 119	51·1 (9·7)	50·3–51·9	27 449	58·3%	56·5–60·0	3464	7·4%	6·3–8·5
Philippines	4767	52·7 (9·6)	51·9–53·5	3401	71·3%	66·9–75·4	302	6·3%	5·4–7·4
Colombia	7499	50·8 (9·6)	50·2–51·4	4808	64·1%	61·1–67·0	282	3·8%	3·0–4·7
Iran	6013	48·5 (9·2)	47·5–49·5	3137	52·2%	44·4–59·8	359	6·0%	5·1–6·9
Occupied Palestinian territory	1644	49·2 (9·6)	48·6–49·9	803	48·8%	47·6–50·1	113	6·9%	5·5–8·5
Bangladesh	2926	46·0 (9·3)	45·4–46·6	1596	54·5%	52·9–56·2	80	2·7%	2·2–3·4
India	29 165	48·7 (10·4)	48·1–49·2	16 388	56·2%	54·2–58·2	773	2·7%	2·2–3·1
Pakistan	2397	47·4 (9·1)	46·0–48·7	1236	51·6%	47·8–55·3	126	5·3%	2·2–12·0
Zimbabwe	1220	51·4 (10·1)	48·3–54·6	821	67·3%	49·2–81·4	70	5·7%	2·3–13·4
Tanzania	1987	49·9 (11·3)	49·4–50·4	1518	76·4%	68·5–82·8	101	5·1%	2·8–9·0
All countries	166 181	50·6 (9·9)	50·3–50·9	96 528	58·1%	57·2–59·0	8492	5·1%	4·7–5·5

Countries are ordered by descending income level.

* CVD is coronary artey disease or stroke.

The proportion of participants with cardiovascular disease who were taking three or more secondary prevention medications ranged from 0% in South Africa (95% CI 0–1·7), Tanzania (0–3·6), and Zimbabwe (0–5·1), to 49·3% (44·4–54·3) in Canada. The proportions using at least one drug for secondary prevention are higher, but vary significantly from 2·0% (95% CI 0·5–6·9) in Tanzania to 91·4% (86·6–94·6) in Sweden (table 2). The proportion of people with cardiovascular disease using each individual type of cardiovascular disease secondary prevention medicine is shown in table 3.

**Table 2:** Use of secondary prevention medicines among those reporting cardiovascular disease in the PURE study

	**Three or more secondary prevention drugs**			**One or more secondary prevention drugs**		
	N	%	95% CI	N	%	95% CI
Canada	299	49·3%	44·4–54·3	546	90·1%	87·7–92·1
Sweden	72	44·2%	35·2–53·6	149	91·4%	86·6–94·6
United Arab Emirates	28	38·9%	22·2–58·7	61	84·7%	71·8–92·4
Saudi Arabia	17	24·6%	10·8–47·0	50	72·5%	56·7–84·1
Argentina	25	8·5%	4·8–14·7	222	75·8%	70·4–80·5
Brazil	69	16·5%	11·7–22·7	337	80·6%	77·2–83·6
Chile	11	9·6%	2·1–34·2	70	60·9%	29·1–85·5
Malaysia	33	7·6%	4·3–13·1	152	34·9%	25·1–46·3
Poland	35	26·7%	22·7–31·2	112	85·5%	80·1–89·6
South Africa	0	0·0%	0·0–1·7	56	26·4%	16·2–40·0
Turkey	37	12·0%	9·3–15·5	195	63·3%	56·2–69·9
China	32	0·9%	0·6–1·4	1435	41·4%	36·7–46·3
Philippines	7	2·3%	1·1–4·6	186	61·6%	53·3–69·2
Colombia	30	10·6%	6·6–16·7	150	53·2%	45·0–61·2
Iran	69	19·2%	14·5–25·1	263	73·3%	67·9–78·0
Occupied Palestinian territory	23	20·4%	13·2–30·1	98	86·7%	79·1–91·9
Bangladesh	1	1·3%	0·2–8·8	14	17·5%	9·0–31·3
India	19	2·5%	1·0–5·7	186	24·1%	16·8–33·2
Pakistan	1	0·8%	0·1–9·5	34	27·0%	5·0–72·3
Zimbabwe	0	0·0%	0·0–5·1	22	31·4%	5·9–76·9
Tanzania	0	0·0%	0·0–3·6	2	2·0%	0·5–6·9

Countries are ordered by descending income level.

**Table 3:** Use of secondary prevention medications among participants reporting a cardiovascular disease in the PURE study

	**N**	**Antiplatelet drugs**			**ACE inhibitors or ARBs**			**β-blockers**			**Lipid-lowering drugs**			**Calcium-channel blockers**			**Diuretics**		
		n	%	95% CI	n	%	95% CI	n	%	95% CI	n	%	95% CI	n	%	95% CI	n	%	95% CI
Canada	606	409	67·5	62·4–72·2	349	57·6	52·3–62·7	237	39·1	34·9–43·5	447	73·8	69·2–77·8	130	67·5	62·4–72·2	117	57·6	52·3–62·7
Sweden	163	119	73·0	66·8–78·4	58	35·6	27·6–44·5	89	54·6	47·0–62·0	102	62·6	51·5–72·5	39	73·0	66·8–78·4	22	35·6	27·6–44·5
UAE	72	47	65·3	40·7–83·7	27	37·5	18·7–61·0	24	33·3	21·2–48·2	41	56·9	43·7–69·2	16	65·3	40·7–83·7	5	37·5	18·7–61·0
Saudi Arabia	69	36	52·2	34·9–69·0	13	18·8	8·9–35·6	20	29·0	14·4–49·8	28	40·6	25·2–58·1	16	52·2	34·9–69·0	6	18·8	8·9–35·6
Argentina	293	97	33·1	25·0–42·4	135	46·1	41·3–51·0	126	43·0	36·9–49·4	52	17·7	13·3–23·2	37	33·1	25·0–42·4	47	46·1	41·3–51·0
Brazil	418	138	33·0	26·9–39·7	195	46·7	39·9–53·5	160	38·3	32·6–44·3	118	28·2	20·0–38·2	68	33·0	26·9–39·7	135	46·7	39·9–53·5
Chile	115	45	39·1	17·5–66·0	44	38·3	17·9–63·7	21	18·3	5·1–48·1	21	18·3	4·6–50·7	10	39·1	17·5–66·0	22	38·3	17·9–63·7
Malaysia	435	62	14·3	9·1–21·7	46	10·6	5·7–18·8	45	10·3	6·0–17·3	66	15·2	9·0–24·5	47	14·3	9·1–21·7	27	10·6	5·7–18·8
Poland	131	63	48·1	30·1–66·6	60	45·8	36·3–55·6	58	44·3	35·4–53·6	58	44·3	29·8–59·8	25	48·1	30·1–66·6	24	45·8	36·3–55·6
South Africa	212	13	6·1	3·2–11·5	20	9·4	2·0–34·2	8	3·8	1·0–12·9	3	1·4	0·2–11·8	11	6·1	3·2–11·5	24	9·4	2·0–34·2
Turkey	308	100	32·5	26·3–39·3	101	32·8	27·9–38·1	96	31·2	25·3–37·7	63	20·5	16·1–25·6	40	32·5	26·3–39·3	75	32·8	27·9–38·1
China	3464	587	16·9	13·9–20·5	267	7·7	6·5–9·1	191	5·5	4·3–7·1	77	2·2	1·5–3·2	458	16·9	13·9–20·5	440	7·7	6·5–9·1
Philippines	302	23	7·6	3·8–14·7	101	33·4	22·3–46·8	30	9·9	4·4–20·8	50	16·6	12·3–22·0	81	7·6	3·8–14·7	1	33·4	22·3–46·8
Colombia	282	82	29·1	21·6–37·9	77	27·3	21·8–33·6	47	16·7	11·8–23·1	51	18·1	13·7–23·6	29	29·1	21·6–37·9	34	27·3	21·8–33·6
Iran	359	170	47·4	41·0–53·8	72	20·1	15·0–26·3	159	44·3	39·7–49·0	112	31·2	24·9–38·3	60	47·4	41·0–53·8	40	20·1	15·0–26·3
OPT	113	69	61·1	50·4–70·8	39	34·5	25·4–44·9	33	29·2	21·5–38·3	41	36·3	26·3–47·6	21	61·1	50·4–70·8	25	34·5	25·4–44·9
Bangladesh	80	3	3·8	1·2–11·3	4	5·0	1·5–15·6	6	7·5	2·9–17·8	2	2·5	0·6–9·8	1	3·8	1·2–11·3	2	5·0	1·5–15·6
India	773	78	10·1	6·2–16·0	42	5·4	3·7–8·0	91	11·8	7·1–19·0	34	4·4	2·4–7·9	54	10·1	6·2–16·0	17	5·4	3·7–8·0
Pakistan	126	24	19·0	4·0–57·2	6	4·8	0·5–32·6	11	8·7	1·5–37·7	6	4·8	1·5–14·2	8	19·0	4·0–57·2	3	4·8	0·5–32·6
Zimbabwe	70	4	5·7	0·4–50·6	4	5·7	0·1–71·3	1	1·4	0·1–27·6	0	0·0	0·0–5·1	6	5·7	0·4–50·6	16	5·7	0·1–71·3
Tanzania	101	1	1·0	0·2–4·4	1	1·0	0·2–4·4	0	0·0	0·0–3·6	0	0·0	0·0–3·6	1	1·0	0·2–4·4	0	1·0	0·2–4·4

Countries are ordered by descending income level. ACE=angiotensin-converting enzyme. ARB=angiotensin-receptor blocker. UAE=United Arab Emirates. OPT=occupied Palestinian territory.

**Figure 1:**
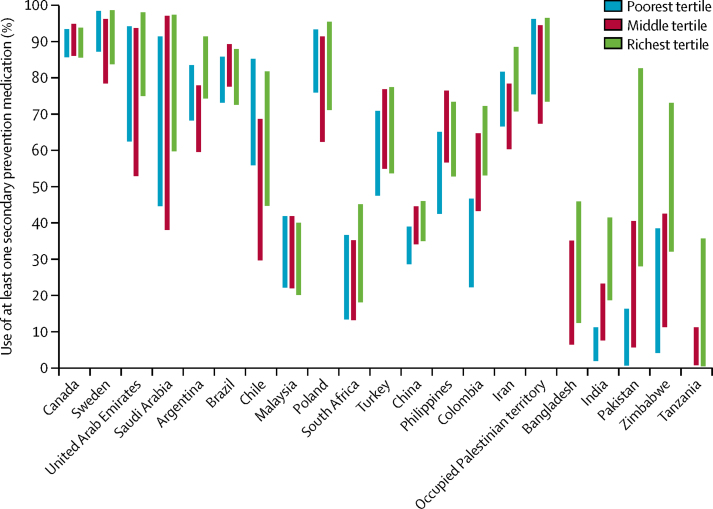
High-low plot showing the 95% confidence range for the use of at least one secondary prevention drug by wealth tertile in the PURE study countries Countries are ordered by 2006 per capita gross domestic product. Countries with significant p values for the hypothesis test that the absolute difference in adjusted prevalence between the richest and poorest tertiles is equal to zero: China, p=0·0259; Colombia, p=0·0002; Bangladesh, p=0·0025; India, p=0·0000; Pakistan, p=0·0006; and Zimbabwe, p=0·0066.

From our sample of those with cardiovascular disease, 5·7% of individuals were missing data on household wealth. These individuals were excluded from inequality analyses and information. Missing data on household wealth by country is shown in the appendix (p 9). All other variables used in the analysis were complete. The proportion of individuals using at least one drug among those with cardiovascular disease was higher in the richest wealth index tertile than in the poorest tertile in all countries except Canada, Sweden, Brazil, Chile, Poland, Malaysia, and the occupied Palestinian territory, where it was either similar in the richest and poorest groups, or higher among the poorest. In Pakistan, use of at least one drug was 18·6 times higher among the richest tertile than among the poorest tertile (57·7% *vs* 3·1%); in India, it was 6·2 times higher (28·7% *vs* 4·6%), and in Zimbabwe 3·8 times higher (53·1% *vs* 13·9%; figure 1).

Wagstaff's adjusted concentration index values for use of at least one medication are shown in table 4. Negative Wagstaff's adjusted concentration index values indicate greater use among the poor (pro-poor), whereas positive values indicate pro-rich distribution of medication use. There was significant (at the 5% level) pro-rich inequality in Saudi Arabia, China, Colombia, India, Pakistan, and Zimbabwe. Wagstaff's adjusted concentration index values suggested a pro-poor distribution of use of minimum medication for cardiovascular disease in Sweden, Chile, Poland, and the occupied Palestinian territory, but none of these estimates were significant.

**Table 4:** Adjusted Wagstaff concentration indices for use of at least one secondary prevention medication, by country in the PURE study

	**Estimate**	**SE**	**95% CI**
Canada	0·0202	0·0801	−0·1368 to 0·1772
Sweden	−0·0210	0·1563	−0·3274 to 0·2855
United Arab Emirates	0·1979	0·1828	−0·1603 to 0·5562
Saudi Arabia*	0·3278	0·1623	0·0097 to 0·6458
Argentina	0·0320	0·0784	−0·1217 to 0·1857
Brazil	−0·0258	0·0698	−0·1626 to 0·1110
Chile	−0·0749	0·0944	−0·2600 to 0·1102
Malaysia	0·0634	0·0589	−0·0521 to 0·1788
Poland	−0·0592	0·1445	−0·3423 to 0·2240
South Africa	0·1383	0·0920	−0·0420 to 0·3186
Turkey	0·1070	0·0649	−0·0202 to 0·2341
China*	0·1342	0·0200	0·0949 to 0·1734
Philippines	0·1201	0·0706	−0·0182 to 0·2584
Colombia*	0·2187	0·0620	0·0972 to 0·3403
Iran	0·0373	0·0659	−0·0918 to 0·1664
Occupied Palestinian territory	−0·0490	0·1470	−0·3371 to 0·2391
Bangladesh	0·2662	0·1745	−0·0757 to 0·6081
India*	0·4841	0·0516	0·3830 to 0·5853
Pakistan*	0·6231	0·1069	0·4136 to 0·8325
Zimbabwe*	0·3550	0·1507	0·0596 to 0·6504
Tanzania	0·3667	0·4786	−0·5714 to 1·3048

* Statistically significantly pro-rich at 5% level. Positive=pro-rich. Negative=pro-poor.

**Figure 2:**
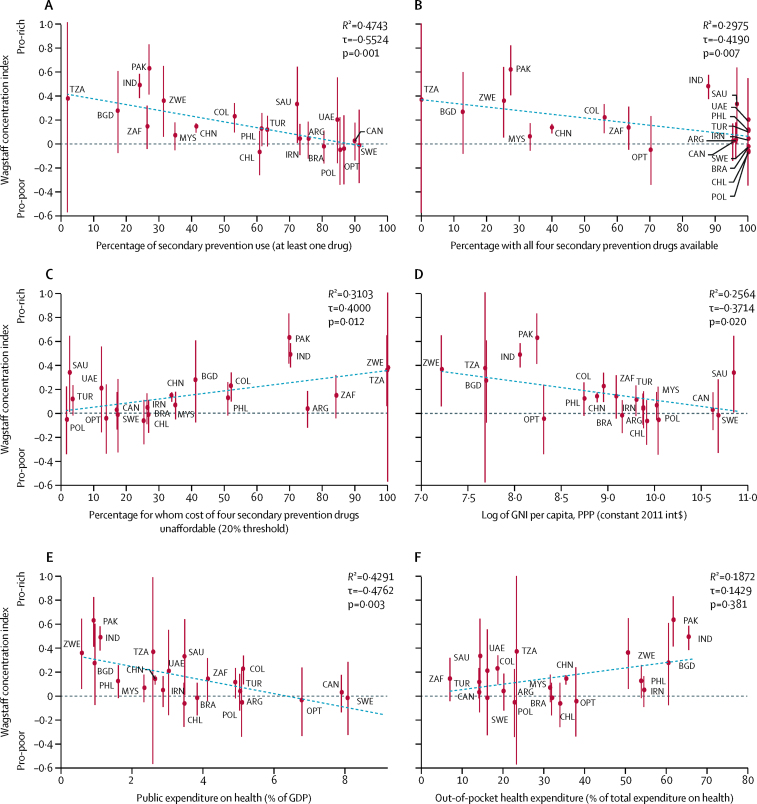
Scatter plots of Wagstaff concentration index of inequality in secondary prevention use against national-level and community-level health system factors (A) Use of at least one secondary prevention drug, (B) availability and (C) affordability of medicines, (D) gross national income per capita, (E) public expenditure on health as a proportion of GDP, and (F) out-of-pocket payment as a proportion of total health expenditure. ARG=Argentina. BGD=Bangladesh. BRA=Brazil. CAN=Canada. CHL=Chile. CHN=China. COL=Colombia. IND=India. IRN=Iran. MYS=Malaysia. OPT=occupied Palestinian territory. PAK=Pakistan. PHL=Philippines. POL=Poland. SAU=Saudi Arabia. SWE=Sweden. TUR=Turkey. TZA=Tanzania. UAE=United Arab Emirates. ZAF=South Africa. ZWE=Zimbabwe. GNI=gross national income. GDP=gross domestic product. PPP=purchasing power parity. Int$=international dollar, adjusted for purchasing power parity.

There is a significant inverse association between the measure of socioeconomic inequality and the proportion of those using at least one secondary prevention drug. Countries that rank higher in terms of average use of at least one cardiovascular disease medication rank lower in the degree to which utilisation is pro-rich (Wagstaff's adjusted concentration index–mean rate: Kendall's tau [τ]=–0·5524; p=0·001; figure 2A). Plots of each country's Wagstaff's adjusted concentration index against availability (τ=–0·4190; p=0·007) and affordability of medicines (τ=0·4000; p=0·012), gross national income per capita (τ=–0·3714; p=0·020), public expenditure on health as a proportion of GDP (τ=–0·4762; p=0·003), and out-of-pocket payment as a proportion of total health expenditure (τ=0·1429; p=0·381) are shown in figure 2B–F. Of the factors plotted, the strongest predictors of variation in inequality among countries are overall secondary prevention use (*R*^2^=0·4743) and public expenditure on health as a proportion of GDP (*R*^2^=0·4291).

## Discussion

To our knowledge, this study is the first to present estimates of the rate of, and inequality in, secondary prevention of cardiovascular disease in individual countries at various levels of development. Our results reveal alarmingly low use of optimal secondary prevention of cardiovascular disease in many countries. Whereas the lowest use was observed in low-income and middle-income countries, specifically South Africa, Tanzania, and Zimbabwe, even the high-income countries included in our study have not reached the modest target of 50% coverage of drug therapy with three of the four drugs set out in the WHO Global Monitoring Framework for Non-Communicable Diseases.^5^ This target includes patients with no previous cardiovascular disease event but at more than 30% risk of experiencing one within 10 years, who are excluded from our study. So, we are likely to underestimate the challenge ahead. Although the low overall rates we observed in high-income countries such as Canada might be surprising, they are consistent with findings from the USA,^23^ and reinforce the need for greater efforts to reach WHO targets for reduction of cardiovascular disease mortality by addressing treatment gaps in all countries, not only in low-income countries.

In several countries in our study, the situation is much worse for the poorest, with significant pro-rich inequality in the use of at least one drug observed in China, Saudi Arabia, Colombia, India, Pakistan, and Zimbabwe. The findings from China are consistent with recent evidence of unaffordability of cardiovascular disease drugs; for example, the cost of a month's supply of a generic brand of atorvastatin (a cholesterol-lowering drug) is equivalent to 6·7 days' wages of the lowest paid government workers.^24^ Another study from China found lower use of secondary prevention among older people with less education and younger people with lower incomes.^12^

We found greater socioeconomic inequality in the use of drugs in countries with lower mean use of secondary prevention medicines. This finding is consistent with the inverse equity hypothesis^25^, ^26^ that medical technologies are initially used to a greater extent by the socially privileged and inequalities only begin to fall once the needs of the rich are met. One potential policy response is to concentrate on raising the average rate of medication use by securing universal access without targeting any particular group. At least in theory, relative disparities should narrow as long as all groups benefit and none fall through the net. This approach is simpler and might be cheaper than identifying those with greatest unmet need. But it is important to ensure that this is working as intended because, otherwise, there is a risk of reinforcing existing gaps in medicine use. By contrast, “progressive universalism”^27^ that targets resources on those with greatest unmet need within a system promoting universal access to essential treatment might achieve the greatest reduction in avoidable deaths while simultaneously reducing inequities.

Left unaddressed, these inequalities in treatment use will ultimately exacerbate inequalities in cardiovascular disease.^28^, ^29^, ^30^ Yet, the inequality we have observed is not inevitable. Our scatter plots provide insight into factors that might explain part of the cross-country variation in inequality. Some variation is explained by availability and affordability of secondary prevention medications in the studied communities. This finding is consistent with our earlier work showing that, although drugs for secondary prevention of cardiovascular disease are licensed and distributed in all countries in this study, in both branded and generic forms, they are less likely to be stocked by retailers based in rural and poor communities.^7^ A further problem is that people living in our rural communities might have to travel much longer distances to reach a pharmacy. It is also consistent with evidence from other low-income and middle-income countries, which suggests that the costs of cardiovascular disease medication are a major contributor to risks of catastrophic medical expenditure and a barrier to treatment, especially among the poorest,^31^, ^32^, ^33^ suggesting that in countries without universal health coverage, poverty negatively affects access to medicines for coronary heart disease.^10^ More research is needed on the extent of the economic burden imposed by chronic treatment costs for cardiovascular disease on poor households in a wider range of countries,^34^ and the impact that this burden has on their decision to adhere to care.

Aside from the mean use of secondary prevention of cardiovascular disease, the strongest predictor of inequality in use was public expenditure on health as a proportion of GDP. A recent report from the Chatham House Centre on Global Health Security^35^ concluded that in order for countries to achieve minimum standards of health-care access and financial protection, public (or government) health expenditure as a proportion of GDP should be at least 5%. Among those countries in our study, this target is only reached in Canada (7·9%), Sweden (8·1%), Argentina (5·0%), Poland (5·1%), Colombia (5·1%), and the occupied Palestinian territory (6·8%; Turkey spends 4·9%).^22^ The target for government spending is only one aspect of a comprehensive financing framework.^35^ It must be combined with policies to ensure that health care is delivered efficiently to those who need it, addressing patient-level barriers, such as lack of health literacy or awareness of the importance of treatment adherence.^10^, ^33^, ^36^ Recent evidence suggests that the polypill (combination pill including three or four of the secondary prevention drug types) might improve adherence.^37^

Brazil might provide one example of a successful policy approach to reducing inequality in preventive and primary care. Most common medications are free at the point of service for all citizens. Additionally, the country's Family Health Strategy uses a community-based approach to improve access to primary health care for previous underserved populations, including extensive use of community health workers to support patients in adhering to medication regimens.^38^ Evidence has shown that the poorest municipalities in Brazil have particularly benefited from the Family Health Strategy^39^ and the programme is associated with reductions in cardiovascular disease mortality and hospitalisations.^40^

Our study has some limitations. First, samples were not selected to be nationally representative and the numbers with cardiovascular disease are low in some countries, limiting scope for disaggregated analyses and resulting in large error margins for our estimates from some countries. While the samples are similar to the national population in respect of major demographic and socioeconomic characteristics,^14^ one criterion for selection of communities was that they facilitated long-term follow-up. Hence, extremes of the economic spectrum, especially the lower bound, are probably excluded. While this will probably lead to underestimates of use of secondary prevention and wealth-related inequality, our estimates should be interpreted with caution and followed up with further country-specific research. As mentioned, a small percentage of our sample of individuals with cardiovascular disease was also excluded from our analysis due to missing data. We do not impute for these missing wealth data and these observations are excluded from our analyses, which assumes that missing wealth data is completely at random in all countries. This assumption might bias our inequality estimates, although the direction of that bias is unclear. Second, our data rely on self-reported coronary heart disease and stroke, and are therefore potentially vulnerable to bias. However, as discussed above, self-reports were verified against medical or hospital records in 455 reported events, with a confirmation rate of 89%, and available data from other studies of stroke and myocardial infarction support the accuracy of self-reports.^41^, ^42^, ^43^, ^44^ Third, our data are cross-sectional and we cannot determine whether participants are prescribed and commenced on secondary prevention and then cease using the medication, or whether they are never prescribed the medication; nor can we tell whether they are using the medication as prescribed (eg, daily *vs* weekly). For example, one study using registry data from India found that about half of all patients suffering a myocardial infarction were discharged on secondary prevention but adherence declined rapidly.^45^ Fourth, quantitative analyses reveal but do not explain socioeconomic inequality. The next step requires multidisciplinary research to understand observed variations, as in earlier studies of hypertension in Malaysia^46^ and Colombia.^47^ Fifth, we do not know the reasons why individuals were using medicines. Thus, some might have been initiated to treat hypertension rather than explicitly for secondary prevention. We might, therefore, be seeing an effect of varying local practices and guidelines for hypertension. For example, in South Africa, initial treatment with diuretics or calcium channel blockers (or both) is recommended for black patients because they are more effective in this population than angiotensin-converting-enzyme inhibitors.^48^ However, regardless of whether the blood-pressure-lowering drug being used by the individual with cardiovascular disease was initially prescribed for hypertension, what is important is that this individual is taking secondary prevention medication, and therefore has the ability to benefit from it. Finally, it is possible that in some countries where health-care reforms have been implemented recently, such as Iran, our data fail to capture resulting increases in secondary prevention use.

Secondary prevention medicines are highly effective in avoiding recurrence of cardiovascular disease events, which can be especially devastating for people living in low-income and middle-income countries where acute, life-saving treatment might not be easily available and the economic consequences of illness are severe. Our findings revealed both remarkably low rates of use of known effective secondary prevention medications in several countries, but also statistically significant inequality in some low-income and middle-income countries. The UN and WHO have now recognised the need to reduce the burden of non-communicable diseases, including cardiovascular diseases, and to narrow inequalities in premature mortality. To realise these goals, increased and more equitable secondary prevention must be high on the agenda.
