# Supplementary material for: Inequalities in the use of secondary prevention of cardiovascular disease by socioeconomic status: evidence from the PURE observational study
Source: Lancet Glob Health. 2018 Feb 9;6(3):e292–301. doi: 10.1016/S2214-109X(18)30031-7 (PMC5905400; doi:10.1016/S2214-109X(18)30031-7)
Supplement: Supplementary appendix [file mmc1.pdf]

THE LANCET
Global Health

Supplementary appendix

This appendix formed part of the original submission and has been peer reviewed. We post it as supplied by the authors.

Supplement to: Murphy A, Palafox B, O'Donnell O, et al. Inequalities in the use of secondary prevention of cardiovascular disease by socioeconomic status: evidence from the PURE observational study. *Lancet Glob Health* 2018; **6:** e292–301.

Appendix

Appendix Table 1: Years of baseline data collection for each country, PURE study
Appendix Table 2: Baseline response rates by country and place of residence, PURE study
Appendix Figure 1: Median, interquartile range and range of wealth index scores by country, PURE study
Appendix Table 3: Erreygers' concentration index for inequality in use of at least one secondary prevention medication, PURE study
Appendix Section 1: Model used to standardize concentration indices
Appendix Section 2: Methods used for the Environmental Profile of a Community's Health (EPOCH) instrument
Appendix Table 4: Utilisation of two or more secondary prevention medications among those with CVD, PURE Study
Appendix Table 5: Missing data on household wealth among CVD sample, by country

Table 1: Years of baseline data collection for each country, PURE study

Country	Years
Canada	2006-2009
Sweden	2005-2009
United Arab Emirates	2006-2009
Saudi Arabia	2012-2014
Argentina	2006-2009
Brazil	2005-2009
Chile	2006-2009
Malaysia	2007-2009
Poland	2007-2009
South Africa	2005-2011
Turkey	2008-2009
China	2005-2010
Philippines	2013-2014
Colombia	2005-2009
Iran	2006-2009
Occupied Palestinian Territory	2012-2013
Bangladesh	2008
India	2002-2007
Pakistan	2009-2011
Zimbabwe	2006-2007
Tanzania	2012-2014

Table 2: Baseline response rates by country and place of residence, PURE study

		Urban	Rural
HIC	Canada	68	72
	Sweden	48	54
	UAE	63	83
MIC	Argentina	64	86
	Brazil	60	77
	Chile	79	89
	China	78	80
	Colombia	70	71
	Iran	76	90
	Malaysia	72	84
	Poland	74	70
	S Africa	69	50
	Turkey	78	84
LIC	Bangladesh	69	95
	India	57	60
	Pakistan	80	81
	Zimbabwe	75	87

Figure 1: Median, interquartile range and range of wealth index scores by country, PURE study

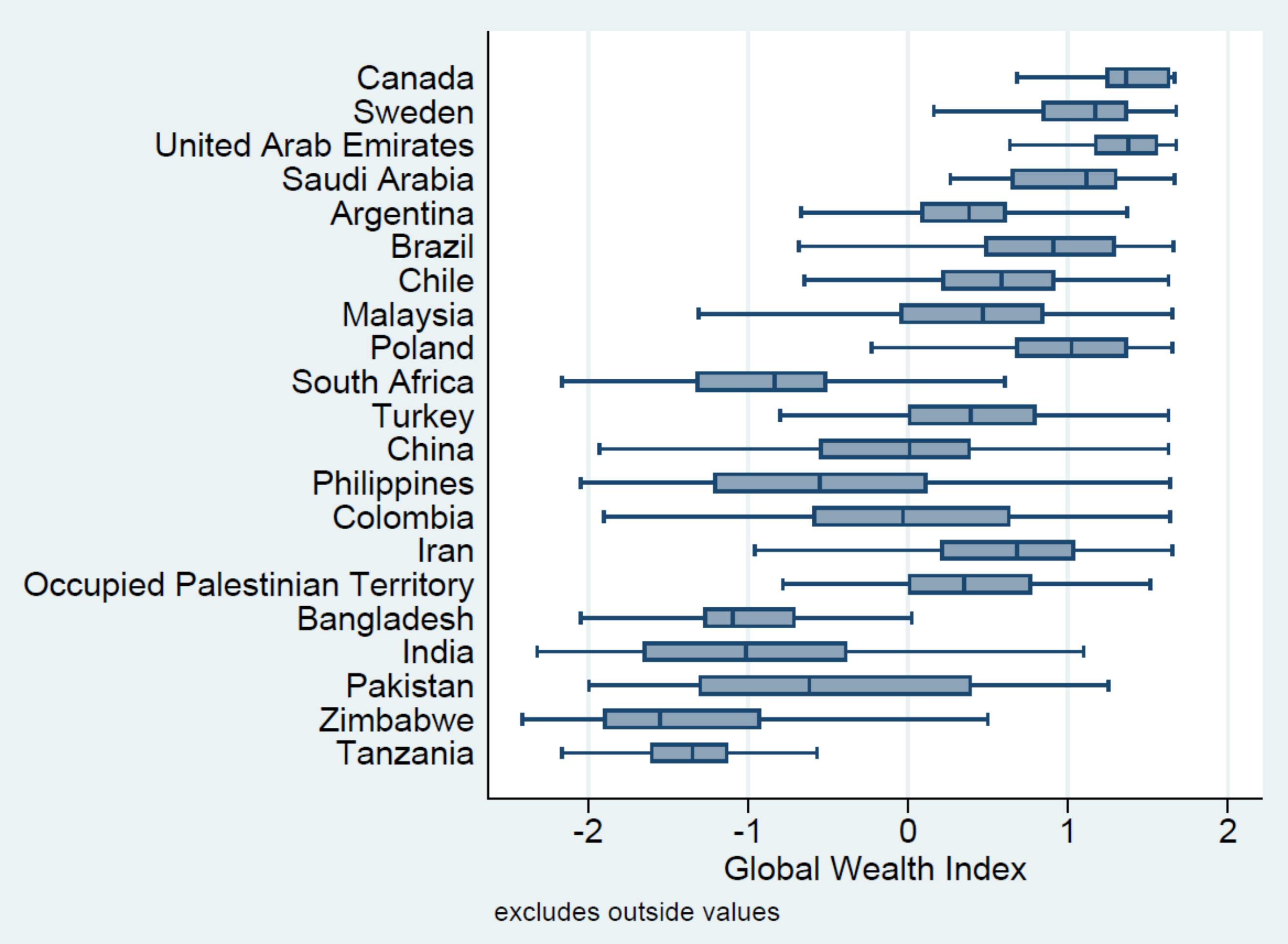

Table 3: Erreygers' concentration index for inequality in use of at least one secondary prevention medication, PURE study

A disadvantage of the Wagstaff concentration index (WCI) used in the paper is that it tends toward extreme values, indicating severe inequality as the prevalence rate becomes either very low or very high. This may be considered paradoxical since, in both situations, the vast majority has the same utilization – almost everyone gets no medication or almost everyone is medicated. For this reason, we checked the robustness of our findings by measuring inequality using Erreygers' CI (ECI), (43) which is simply the covariance between a binary indicator of medication and (fractional) rank in the distribution of the wealth index, multiplied by 8. As before, if there is no socio-economic inequality in use of medication, then the value is zero, while a positive (negative) value indicates inequality in favour of the rich (poor). The ECI will reach a maximum possible value of 1 only if all in the richest half of the CVD sample receive medication, while no one in the poorest half is medicated.

Correlation between WCI and ECI was measured with Kendall's tau rather than Spearman's rho as the former is more accurate with smaller samples.

Estimates of the ECI for use of at least one secondary prevention medication are given in the table immediately below. ECI estimates are highly, but not perfectly, correlated with WCI estimates (Kendall's tau = 0.72; p=0.01). The slight differences in some country rankings by the two indices is due to greater sensitivity of the WCI to the low prevalence of medication in countries such as Tanzania and to the high prevalence in UAE. But the similarity between the estimates and their high degree of correlation confirms that our findings are insensitive to the specific inequality measure used.)

**Erreygers Concentration Index of** inequality in use of at least one secondary prevention medication			
	**Estimate**	**SE**	**p-value**
Canada	0.020	0.028	0.484
Sweden	-0.047	0.055	0.401
UAE	0.082	0.184	0.701
Saudi Arabia	0.041	0.156	0.796
Argentina	0.011	0.030	0.730
Brazil	-0.070	0.033	0.055
Chile	-0.077	0.071	0.336
Malaysia	0.081	0.060	0.185
Poland	0.002	0.049	0.972
South Africa	0.069	0.057	0.256
Turkey	0.085	0.066	0.205
China*	0.109	0.040	0.007
Philippines	0.254	0.180	0.393
Colombia*	0.184	0.072	0.014
Iran	-0.040	0.041	0.334
OPT	-0.082	0.071	0.260
Bangladesh	0.118	0.092	0.205
India*	0.365	0.042	0.000
Pakistan*	0.536	0.074	0.005
Zimbabwe	0.228	0.093	0.134
Tanzania	0.011	0.017	0.511

Section 1: Model used to standardize concentration indices

We obtained the concentration index indirectly standardized for age and sex using the convenient regression approach.

$$2\sigma_r^2\left(\frac{h_i}{\mu}\right) = \alpha_2 + \beta_2 r_i + \sum_j \delta_j x_{ji} + v_i$$

Where h_i is the health sector variable, μ is its mean; r_i is the fractional rank of individual i in the living standards distribution, arranged from the poorest (i=1) to the richest (i=N); σ_r^2 is the variance of the fractional rank; x_{ji} are the confounding variables age, age-square and sex; and $\hat{\beta}_2$ is the OLS estimate of the indirectly standardized concentration index. Note that the concentration index is normalized by dividing through by 1 minus the mean. (14)

Section 2: Methods used for the Environmental Profile of a Community's Health (EPOCH) instrument

The Environmental Profile of a Community's Health (EPOCH) instrument recorded information about community-level factors that can affect cardiovascular disease. Only communities with at least 30 PURE participants were included in EPOCH (90% of PURE communities). This instrument has been shown to be a reliable and feasible indicator of measures of the health environment in diverse settings (15). Data collection methods included an observation walk along a 1 km planned route beginning from a pre-specified central location designated as the starting point (e.g. busy intersections or train station). A pharmacy closest to the starting point was visited to collect information about availability and prices of medicines used to treat hypertension. If a pharmacy was not located within the 1 km observation walk, researchers were instructed to search for a pharmacy located up to 20 km from the starting point from which to gather data. A similar approach was used to collect information about the availability of public and private health care providers within the community.

Table 4: Utilisation of two or more secondary prevention medications among those with CVD, PURE Study

	CVD	Using 2 or more secondary prevention drugs		
	N	N	%	95% CI
Canada	606	477	78.7	74.3-82.5
Sweden	163	125	76.7	69.1-82.9
United Arab Emirates	72	49	68.1	51.0-81.4
Saudi Arabia	69	32	46.4	29.5-64.2
Argentina	293	104	35.5	28.1-43.6
Brazil	418	158	37.8	31.0-45.1
Chile	115	47	40.9	13.6-75.2
Malaysia	435	77	17.7	10.0-29.5
Poland	131	73	55.7	32.9-76.3
South Africa	212	11	5.2	1.3-18.0
Turkey	308	99	32.1	26.1-38.8
China	3464	415	12.0	9.5-15.0
Philippines	302	50	16.6	13.3-20.4
Colombia	282	77	27.3	20.8-34.9
Iran	359	179	49.9	43.5-56.2
Occupied Palestinian Territory	113	62	54.9	44.9-64.4
Bangladesh	80	2	2.5	0.6-9.8
India	773	69	8.9	5.3-14.6
Pakistan	126	17	13.5	2.6-47.2
Zimbabwe	70	2	2.9	0.0-74.4
Tanzania	101	1	1.0	0.2-4.4

Appendix Table 5: Missing data on household wealth among CVD sample, by country

	N (CVD)	Missing wealth	
Canada	606	3	0.5%
Sweden	163	2	1.2%
UAE	72	0	0.0%
S. Arabia	69	2	2.9%
Argentina	293	2	0.7%
Brazil	418	3	0.7%
Chile	115	2	1.7%
Malaysia	435	19	4.4%
Poland	131	0	0.0%
S. Africa	212	12	5.7%
Turkey	308	0	0.0%
China	3,464	319	9.2%
Philippines	302	21	7.0%
Colombia	282	4	1.4%
Iran	359	0	0.0%
OPT	113	8	7.1%
Bangladesh	80	2	2.5%
India	773	73	9.4%
Pakistan	126	3	2.4%
Zimbabwe	70	1	1.4%
Tanzania	101	11	10.9%
**Total**	**8492**	**487**	**5.7%**
